# Kaleidoscopes in the ICU

**DOI:** 10.1017/S1478951526102922

**Published:** 2026-06-11

**Authors:** Tarek Zieneldien

**Affiliations:** The Johns Hopkins University School of Medicine, Baltimore, MD, USA

As I walk into the room, the patient explains that her world has fractured
Into a chapel window,
Reds kneeling beside a blue entrapped in yellow
Every face multiplied like saints
Who refuse to agree on a miracle

In triage, the clock behaves like an empire in decline
Hands circling, borders dissolving,
Minutes annexed by persistent pain
In her chart, the chief complaint said kaleidoscope vision:
It was characterized not by hallucination, but excess truth
Too many angles of the same fear
In her spoken history, the chief complaint was:
Not having enough time
To watch her granddaughter grow up –
whom she met only last month

I think of Hildegard of Bingen at Rupertsberg,
Her Scivias, circles of blinding light and spinning forms
How visions once made women holy
How brightness was mistaken for miracle
Now brightness means pressure
Means mandrakes blooming where they should not

She feels pain, arriving not as invasion, but as inheritance
Cannonballs of cells that remember how to divide,
The way nations remember war
Without instruction on how to stop
Blasting through bone relentlessly
The scans name metastasis, an insatiable hunger

When they wheel her deeper into the ICU,
She leaves behind the fractured saints, the cathedral windows
Here, everything is sterile, snowblinding
Complementing the winter outside the window
A season that never ended,

Still, somewhere behind her eyes,
Colorful orbs riot quietly, refusing to be managed
She tells me that even when her body is failing,
It insists on making art
Out of its own undoing
As if the centuries between saint and symptom have folded,
And every bright fragment has come home to her

